# *Bifidobacterium adolescentis* Strengthens Gut Barrier in Post-Voyage Functional Constipation

**DOI:** 10.3390/ijms262412142

**Published:** 2025-12-17

**Authors:** Huidie Zhao, Hongli Wang, Xinyuan Zhao, Yishan Song, Dong Liang, Yuhao Ma, Zheng Xu

**Affiliations:** 1 Center of Translational Medicine Research, Naval Medical University, Shanghai 200433, China; 2 College of Food Science and Technology, Shanghai Ocean University, Shanghai 201306, China; yssong@shou.edu.cn

**Keywords:** *B. adolescentis*, gut barrier, functional constipation

## Abstract

Prolonged periods of sailing may contribute to the development of functional constipation, which can significantly impair an individual’s work efficiency. Currently, the efficacy of *Bifidobacteria* in treating functional constipation is gaining recognition. However, since the therapeutic effects of *Bifidobacteria* are strain-specific, further research is required on strains isolated from pre-voyage fecal samples. This study examines the role of gut microbiota in post-stroke constipation, aiming to identify specific microbial biomarkers for the development of targeted therapeutic strategies. *B. adolescentis* was identified through metagenomic analysis and subsequently isolated for validation. In the experimental group (EG), C57BL/6J mice received fecal suspension treatment following a 12-day navigation period, which was subsequently followed by a 12-day oral administration of *B. adolescentis*. After treatment, EG significantly improved fecal volume, intestinal motility, and goblet cells; reversed microbial ecological imbalance; reduced pathogens (*E. coli* and *Klebsiella*) by restoring arginine/bile acid metabolism, decreasing Tauro-ursodeoxycholic acid (TUDCA) content, 5-Hydroxytryptamine 4 Receptor (5-HT4R)/*Slc8a1* signaling, and Ca^2+^ signaling pathway; and restoring beneficial species (*B. adolescentis*, *Pseudomonas aeruginosa*). This study provides new insights into probiotics in improving human intestinal health.

## 1. Introduction

Extended periods of maritime residence predispose individuals to various health disorders attributable to environmental, dietary, and other occupational factors. Epidemiological investigations have identified gastrointestinal, musculoskeletal, and cardiovascular conditions as prevalent morbidity patterns among long-term seafarers, with gastrointestinal disorders representing 21% of all reported cases [1]. In gastrointestinal diseases, especially the emergence of functional constipation, seriously affects the quality of life of long-distance voyagers [2]. Severe cases even present with other gastrointestinal and psychological diseases [3]. Patients with severe functional constipation often have concurrent gastroparesis, irritable bowel syndrome (IBS), and functional dyspepsia, which are upper gastrointestinal functional disorders. In the field of mental disorders, we clearly state that the incidence rates of anxiety, depression, and somatization symptoms in these patients are significantly higher than those in the general population and are particularly closely related to generalized anxiety disorder (GAD) and major depressive disorder (MDD). The etiology of functional constipation is complex, which is related to personal behavior and living habits, psychology, environment, age, exercise, sleep, and other factors [4]. Long distance navigation personnel have been in the environment of continuous high temperature, high humidity, noise, and electromagnetic radiation for a long time, resulting in long-term lack of sleep for long-distance navigation personnel [5]. Sleep quality will also decline due to seasickness, crossing time zones, and other reasons. At the same time, studies have found that long-term exposure to these environments also makes long-distance navigators more likely to have symptoms of functional constipation [6,7]. In addition, compared with the land, the marine resources are scarce, and the diet is single. During the long-distance voyage, the vegetables rich in dietary fiber cannot be supplied in time, resulting in insufficient intake, and the structure of intestinal flora changes in different dietary structures [8]. Studies have shown that the abundance of *Holdemanella* and *Plesiomonas* at the level of intestinal flora increased significantly after a long voyage [9]. Another study also found that the abundance of harmful bacteria such as *Klebsiella* in the gut of long-distance voyagers increased after long-distance voyages [10]. Changes in the composition and structure of intestinal flora directly affect intestinal diseases [11]. Therefore, more and more researchers focus on improving the structure of intestinal flora to treat intestinal diseases, especially probiotic products. In addition, it also participates in the synthesis of other beneficial small molecule compounds, affects the transformation process of bile acids, and thus regulates the metabolism of the host, which is of great significance for promoting the health of the host [12,13]. The structure of intestinal flora of long-distance voyagers has changed, and the intake of probiotics can improve the abundance ratio of beneficial and harmful bacteria of intestinal flora to alleviate intestinal diseases [14]. In Ding’s study (2024) on the treatment of adult functional constipation with probiotic products, it was found that probiotic products can improve constipation by increasing the frequency of defecation and increasing the abundance of specific bacteria [15]. *Bifidobacterium*, as a beneficial bacterium in the intestine, can improve the intestinal microenvironment and enhance the body’s immunity. It has been found that the *abfa* cluster contained in *Bifidobacterium longum* can alleviate functional constipation by increasing the utilization of arabinose by intestinal microbes [16]. In the study of Wang (2022), it was found that *Bifidobacterium longum* can increase the content of acetate in the intestine and reduce the expression of AQP8 in the colon to relieve constipation in mice [17].

At present, the research on the mechanism of probiotics in the treatment of functional constipation mainly focuses on 5-HT. Beneficial small molecule compounds that have the function of regulating bile acid transformation include ursodeoxycholic acid (UDCA), oleyl bile acid (OCA), and certain microbial metabolites such as deoxycholic acid (DCA) and lithocholic acid (LCA). Serotonin is a neurotransmitter, mainly distributed in the intestine, secreted, and stored by intestinal chromaffin cells. Although 5-HT itself cannot pass through the blood–brain barrier, the 5-HT derived from the intestine can indirectly affect the central nervous system function by acting on the intestinal vagus nerve endings or regulating intestinal immune cells, thereby participating in the regulation of the gut–brain axis [18]. The 5-HT also stimulates and promotes intestinal motility and vasodilation. [19]. Studies have demonstrated that 5-HT4R agonists can promote intestinal transport and increase fecal water content to relieve constipation [20]. In relevant studies on constipation, it was found that a variety of methods to treat constipation can improve constipation through 5-HT4R. Zhu (2022) found that intestinal flora can increase intestinal stem cells at the base of intestinal crypts and improve the proliferation of intestinal epithelial cells through 5-HT [21]. Wang (2024) found that Torreya grandis seed oil could alleviate loperamide-induced constipation by upregulating the expression of 5-HT4R and other proteins [22]. Similarly, different *Bifidobacteria* genera have differences in the mechanism of treating constipation [23]. Current evidence demonstrates that *Bifidobacterium* alleviates functional constipation through modulation of gut microbiota and enhancement of intestinal 5-HT levels. However, therapeutic efficacy exhibits significant strain-specific variation, particularly in *B. adolescentis* interventions [24]. Multiple studies have shown that probiotic intervention can significantly increase the abundance of beneficial bacteria such as *bifidobacteria* and *lactobacilli* while inhibiting the proliferation of potential pathogenic bacteria such as *Escherichia coli* and *Clostridium* species, thereby improving the balance of intestinal microecology.

Metagenomic sequencing analysis revealed a significant reduction in intestinal *B. adolescentis* abundance following prolonged maritime expeditions. Therefore, this study is based on the study of *B. adolescentis* isolated before the voyage to verify whether *B. adolescentis* is a biomarker of constipation after the voyage. Meanwhile, the mechanism of *B. adolescentis* in the treatment of constipation is still unclear. Transcriptomics and metabolomics were used to explore the mechanism of *B. adolescentis* in the treatment of functional constipation in long-distance voyagers. Long-term seafarers exposed to compound stressors including high temperatures, humidity, noise, vegetable deficiency, and disrupted circadian rhythms exhibit significantly higher rates of functional constipation compared to land-based populations [6,7]. Metagenomic analyses reveal the most pronounced post-voyage decline in gut *Bifidobacterium adolescentis* abundance [9], yet its role as a constipation biomarker and underlying therapeutic mechanisms remain unexplored. This study employs pre- and post-voyage self-controls to identify this bacterium, validates its therapeutic efficacy in animal models, and elucidates its action pathways through transcriptomic and metabolomic analyses. These findings provide precise intervention targets for seafarers.

## 2. Results

### 2.1. Metagenomic Analysis of Species

**Figure 1:**
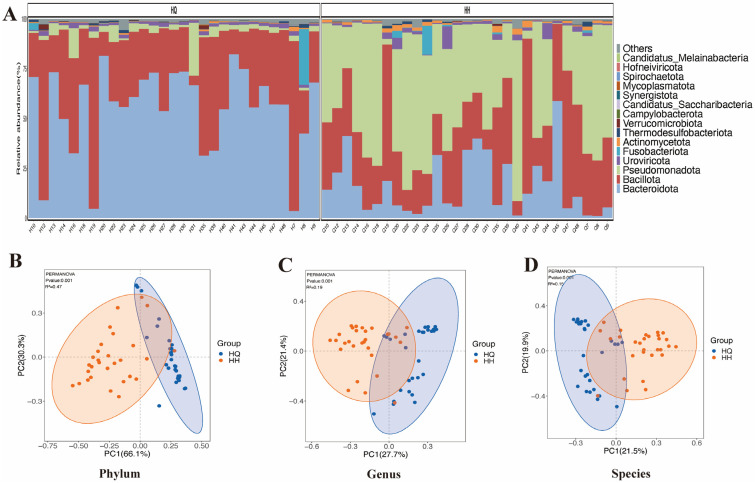
Differences in species community structure before and after the voyage. Panel (**A**) is a histogram of species abundance at the phylum level between the two groups. PCoA plots at the phylum, genus, and species levels are (**B**), (**C**), and (**D**), respectively. HQ: before long voyage; HH: after long voyage.

The microbial composition of the two groups of samples at the phylum level is presented in Figure 1A. The analysis reveals that the predominant phyla include Bacteroidota, Bacillota, Pseudomonadota, Uroviricota, and Fusobacteriota. Notably, Bacteroidota and Bacillota dominate the microbial community prior to the voyage. However, following the voyage, a significant shift is observed, characterized by an increased abundance of *Pseudomonadota* and *Bacteroidota*. In the community distribution abundance index, after long-distance voyages, according to ace calculations, it was found that the community fecal diversity of long-distance voyage personnel was significantly reduced, as shown in Table 1. At the phylum, genus, and species levels, there were significant differences in the species composition of intestinal microbes before and after long-distance voyages. It is comprehensively explained that the species community structure has changed after long-distance voyage (Figure 1B–D). This indicates a decrease in the number of beneficial bacteria, and the trend of constipation is more pronounced.

**Figure 2:**
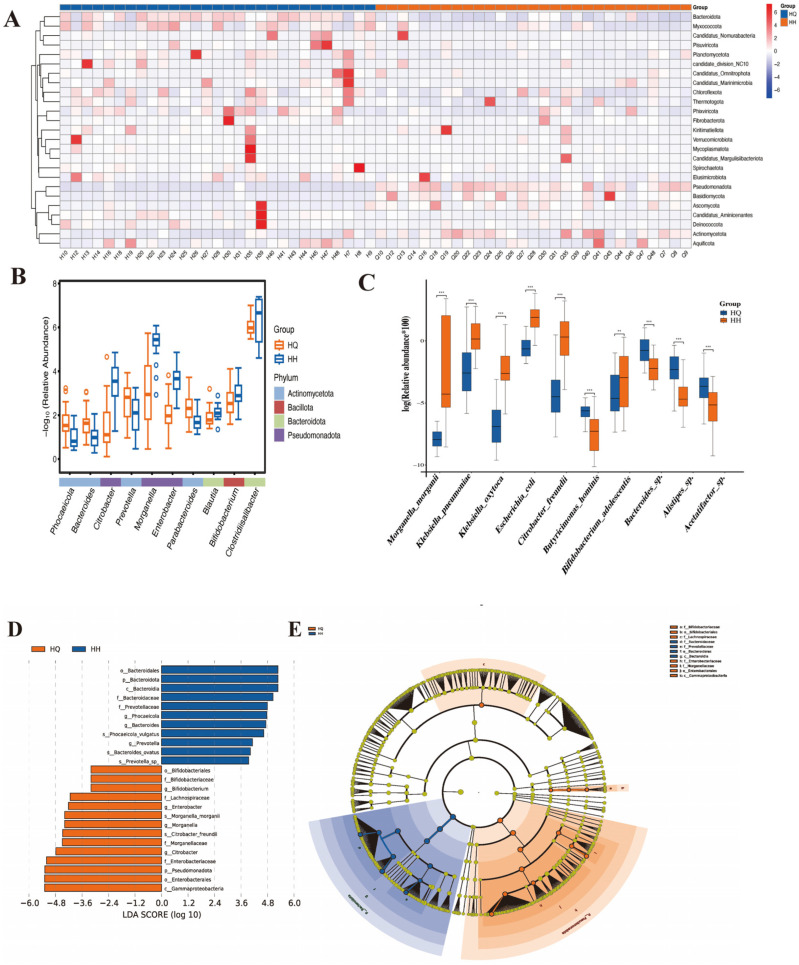
Species level differences the long-term voyage. (**A**) Shows the heat map at the gate level between the two groups of samples. (**B**,**C**) Show box plots with significant differences at the genus level and species level, respectively. ** *p* < 0.01, *** *p* < 0.001. (**D**,**E**) Show the LEfSe results.

According to the Wilcoxon analysis, at the gate level (Figure 2A), the abundances of *Bacteroidota*, *Phixviricota*, *Mycoplasmatota*, and *Spirochaetota* were significantly reduced, and the abundances of *Pseudomonadota*, *Actinomycetota*, and *Basidiomycota* were significantly increased compared to before the voyage. At the genus level, the abundances of *Bifidobacterium*, *Blautia*, *Citrobacter*, *Enterobacter*, and *Morganella* decreased significantly compared to before the voyage, and the abundances of *Bacteroides*, *Clostridiisalibacter*, *Parabacteroides*, and *Prevotella* increased significantly (Figure 2B). At the species level, the abundance of *B. adolescentis*, *Acetatifactor*, *Butyricimonas*, and *Bacteroides* sp. decreased significantly, and *Escherichia*_*coli* and *Morganella*_*morganii*. The abundance of *Klebsiella*_*pneumoniae* and *Klebsiella*_*oxytoca* was significantly increased (Figure 2C).

It was found that the marker flora before and after the voyage changed (Figure 2D,E). Among them, *Bifidobacterium*, *Enterobacter*, *Morganella*, and *Citrobacter* are the sign flora before the voyage, while *phocaeicola*, *Bacteroides*, and *Prevotella* are the biomarkers after the voyage. It can be found that after a long voyage, the human body’s marker flora has changed from probiotics to pathogenic bacteria.

### 2.2. Metagenomic Analysis of Function

At the KEGG level, it was found that after a long voyage, the abundance of pathways related to sugars (N-glycan biosynthesis (ko00510) N-glycan biosynthesis) decreased, and the abundance of pathways involved in membrane transport and signaling increased (Figure S1). After CAZY functional analysis, it was found that glycoside hydrolases (GH), Glycosyl transferases (GT), and carbohydrate esterases (CE) are the main causes of constipation after a long voyage (Figure S2).

**Figure 3:**
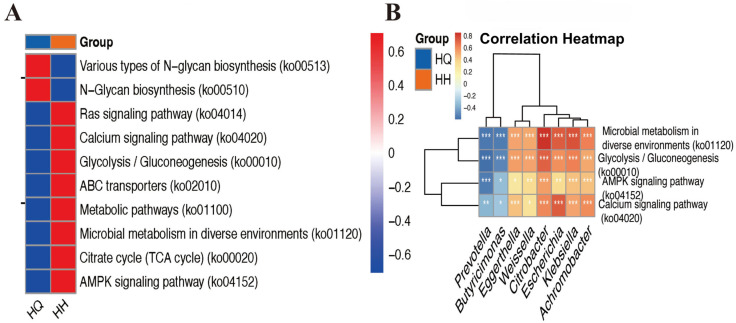
KEGG and CAZy function annotation data analysis diagram. (**A**) Is the heat map of the difference between the two groups at KEGG level 3. (**B**) Shows the correlation analysis between KEGG level 3 and related strains. * *p* < 0.05, ** *p* < 0.01, *** *p* < 0.001.

Analysis of metabolic pathways and the correlation between various enzymes and strains showed the decreased abundance of the AMPK signaling pathway (ko04152), and the calcium signaling pathway (ko04020); GH and other enzymes were negatively correlated with the abundance of *Butyricimonas* and *Prevotella* and positively correlated with the abundance of *Achromobacter*, *Eggerthella*, *Escherichia*, and *Klebsiella* (Figure 3A). Following prolonged voyages, gut microbiota exhibit “reduced glucose metabolism alongside heightened membrane transport and signalling activity”, with this alteration being associated with constipation; concurrently, reduced activity in energy (AMPK) and calcium signaling pathways, alongside alterations in the abundance of relevant glycosylating enzymes (e.g., GH), exhibited negative correlations with specific bacterial genera (*Butyricimonas* and *Prevotella*, etc.), while showing positive correlations with potentially opportunistic pathogens (*Achromobacter*, *Eggerthella*, *Escherichia*, and *Klebsiella*) [25]. This suggests that microbial dysbiosis may represent a key mechanism underlying voyage-related constipation.

### 2.3. B. adolescentis Improves Constipation Parameters

**Figure 4:**
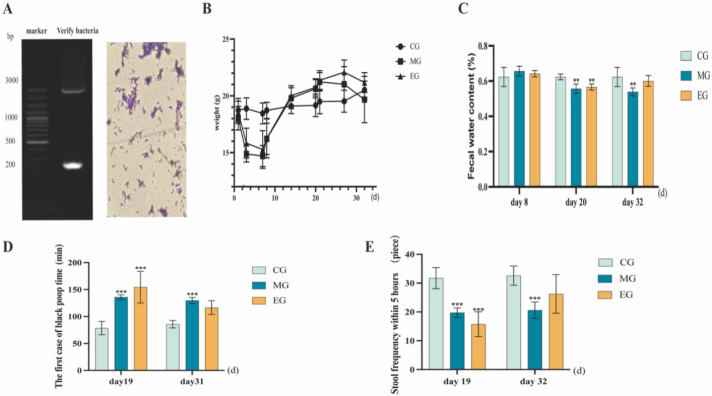
*B. adolescentis* identification and animal experiment results. (**A**) Shows the microscopic examination of *Bifidobacterium adolescentis* after Gram staining (40×). (**B**) Shows the weight trend of mice during modeling. (**C**–**E**) Statistical graphs of fecal water content, the time of the first case of melena, and fecal frequency within 5 h of each group of mice. ** *p* < 0.01, *** *p* < 0.001.

Gram staining of pre-voyage fecal isolates revealed Gram-positive (purple) bacteria. PCR amplification yielded a 200 bp fragment, and sequencing showed 99.66% similarity with *B. adolescentis*. Therefore, the isolated strain was *B. adolescentis* (Figure 4A).

Following 7-day broad-spectrum antibiotic administration, mice exhibited significant weight reduction. Subsequent constipation model establishment revealed gradual weight gain in both model (MG) and experimental (EG) groups. Notably, *B. adolescentis* supplementation reversed this trend, resulting in decreased body weight. As shown in Figure 4B, initial fecal water content did not differ significantly between groups. The CG, MG, and EG exhibited significantly lower fecal water content, delayed black stool onset, and reduced 5-h defecation frequency. After 12 days, the MG group still maintained a lower stool water content, longer black feces appeared, and less stool frequency within 5 h. In the EG, the stool water content and stool frequency increased after gavage with *B. adolescentis*, and the time of melena in the first case became shorter. (Figure 4C–E). *B. adolescentis* significantly alleviates antibiotic-induced gut microbiota dysbiosis and constipation phenotypes—specifically manifested by restoring fecal water content, shortening the time to first black stool passage, and increasing the frequency of bowel movements within five hours, while also exhibiting an inhibitory effect on abnormal weight gain.

### 2.4. B. adolescentis Increased the Number of Goblet Cells and 5-ht4r in the Colon

**Figure 5:**
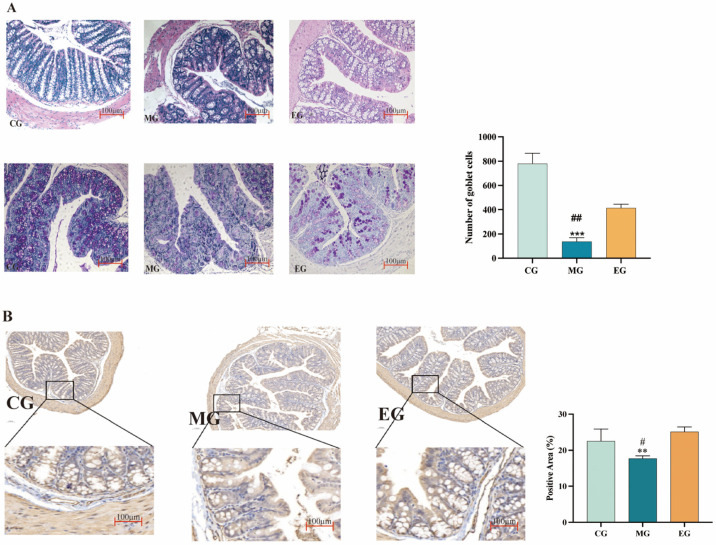
H&E staining sections, PAS staining, statistical analysis of mice in each group, immunohistochemical sections, and statistical analysis of mouse colon tissue. (**A**) Colon transection H&E and PAS, and the results of statistical analysis of slice data. (**B**) Immunohistochemical section of mouse colon tissue (10×), and the statistical analysis of the proportion of positive area in each group. ^#^ *p* < 0.05 compared with CG, ^##^ *p* < 0.01 compared with CG, ** *p* < 0.01 compared with EG, *** *p* < 0.001 compared with EG.

The number of goblet cells in the MG group was significantly reduced, and the number of goblet cells in the colon of mice was increased after treatment with *B. adolescentis* (Figure 5A). The results of immunohistochemistry were used to analyze the expression level of 5-HT4R in colon tissues of mice in each group, and the expression level of 5-HT4R in the EG was significantly lower than that in the MG group compared with that in the CG group, and the expression level of 5-HT4R in the EG was significantly higher than that in the MG group after the ingestion of *B. adolescentis*. Figure 5B demonstrates that *B. adolescentis* reverses mucosal secretion and neural signaling defects in a constipation model by restoring goblet cell numbers and upregulating colonic 5-HT4R expression, thereby improving intestinal motility.

### 2.5. Transcriptome Analysis Results

Transcriptomic analysis of mouse colon tissue samples from CG, MG, and EG showed that a total of 492 differentially expressed genes were obtained in CG and MG, 104 of which showed high expression and 388 showed low expression. There were 266 differentially expressed genes in MG and EG; a total of 80 showed high expression, and 186 showed low expression. *B. adolescentis* significantly modulates colonic gene expression profiles, alleviating constipation-associated transcriptional dysregulation.

**Figure 6:**
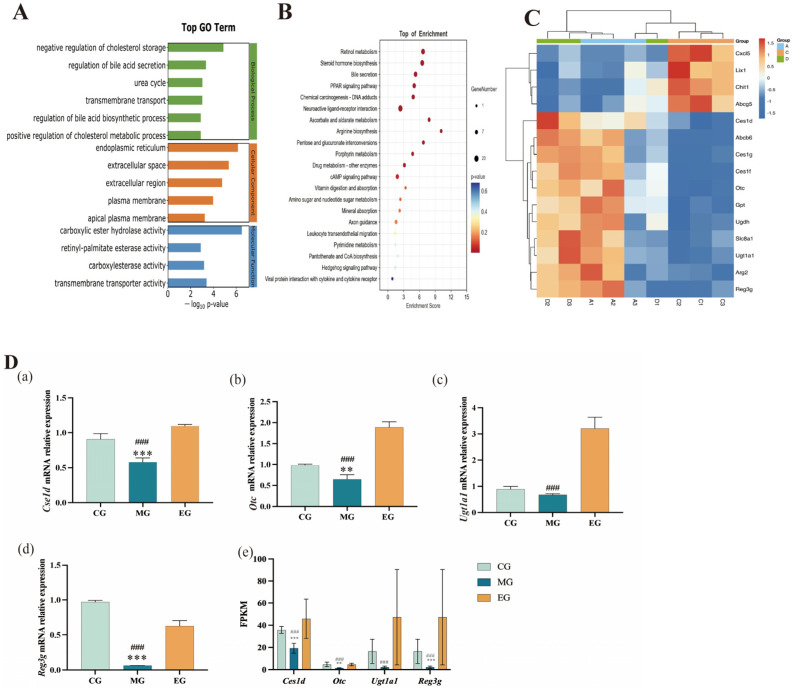
Transcriptomics analysis results. (**A**) Shows the results of go enrichment analysis; (**B**) shows KEGG enrichment top20 bubble diagram; (**C**) shows the clustering diagram of common differential genes in CG, MG and EG; (**D**) shows the statistical chart of colon transcriptome analysis. (**a**) *Ces1d*, (**b**) *Oct*, (**c**) *Ugt1a1*, (**d**) *Reg3g* genes fluorescence quantitative verification of mRNA. (**e**) The FPKM statistical results of the key gene transcriptome data. ** *p* < 0.01 compared with CG; *** *p* < 0.001 compared with CG. ^###^ *p* < 0.001 compared with EG.

GO enrichment analysis was carried out for CG vs. MG and MG vs. EG, and a total of 21 genes were different (Figure 6A). Among them, 12 biological processes (BP), 5 cellular components (CC), and 4 molecular functions (MF) are involved. Focusing on BP, we can find that it is mainly related to the regulation of bile acid secretion and transmembrane transport. According to the KEGG pathway analysis results, there are 21 common differential pathways, which are found to be mainly related to the metabolism of arginine and other substances (pentose and gluconate interconversion of pentose and glucuronide) and the conversion of sugars (amino sugar and nucleotide sugar metabolism) (Figure 6C). *Ces1d*, *Slc8a1*, and *Reg3b* are highly correlated with bile acid metabolism, transmembrane transport, and arginine metabolism, respectively (Table 2 and Figure 6B). Strain *B. adolescentis* reverses colonic transcriptional dysregulation via the “bile acid–glycometabolism–barrier immunity” network.

According to the discovery that *B. adolescentis* can affect the bile acid metabolism pathway, the bile acid content in the intestinal contents of mice was detected. The colon tissues of mice were collected for quantitative gene detection. The results showed that compared with the CG, the relative expression of *Ces1d*, *Otc*, *Ugt1a1*, and *Reg3g* mRNA in the colon of mice gavaged with fecal bacteria suspension after a long voyage was significantly reduced (*p* < 0.05); Compared with the MG group, the mRNA expression levels of *Ces1d*, *Otc*, *Ugt1a1*, and *Reg3g* in the colon tissue of mice were significantly up-regulated after intragastric administration of *B. adolescentis* suspension (*p* < 0.05) (Figure 6D). *B. adolescentis* reverses the downregulation of bile acid metabolism and mucosal defense genes induced by long-term exposure to fecal bacteria suspensions by significantly upregulating colonic expression of *Ces1d*, *Otc*, *Ugt1a1*, and *Reg3g*. This directly demonstrates its core mechanism of alleviating constipation through “restoring bile acid signalling to enhance barrier function”.

The transcriptomic signature suggested bile acid metabolism contributes to post-voyage constipation. Subsequent targeted bile acid analysis of intestinal samples was conducted to determine the specific mechanistic relationships. The abundances of tauroursodeoxycholic acid (TUDCA) and taurodeoxycholic acid (TDCA) in the feces of mice after fecal bacteria transplantation after a long voyage were significantly decreased (*p* < 0.05), while the abundances of hyodeoxycholic acid (HDCA) and dehydrolithocholic acid were significantly increased (*p* < 0.05). However, *B. adolescentis* treatment can improve the increased abundance of ursocholic acid (UCA) and dehydrolithocholic acid caused by constipation (Table 3).

### 2.6. The Impact of Protein Expression on the Intestinal Barrier

**Figure 7:**
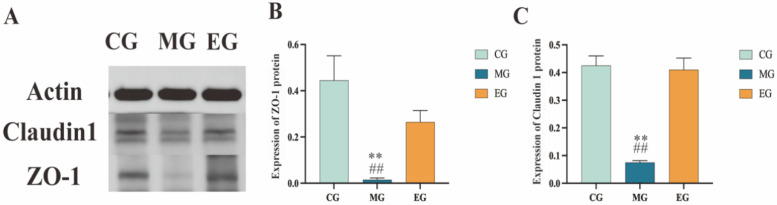
ZO-1 and Claudin1 Protein expression levels. (**A**) ZO-1 and Claudin1 Western Blot bands. (**B**) Statistical analysis of ZO-1 protein bands using Image J. (**C**) Statistical analysis of Claudin-1 protein bands using Image J. ** *p* < 0.01 compared with CG. ^##^ *p* < 0.01 compared with EG.

As shown in Figure 7, the protein expression levels of ZO-1 and Claudin-1 in the intestinal tissue of the MG group were significantly lower than those in the CG group (*p* < 0.05). After *B. adolescentis* intervention, the expression levels of ZO-1 and Claudin-1 significantly increased compared to the MG group (Figure 7A–C). *B. adolescentis* significantly restores the suppressed expression of tight junction proteins ZO-1 and Claudin-1 in constipation models, suggesting it exerts its anti-constipation effects by repairing the integrity of the intestinal mucosal barrier.

## 3. Discussion

*Bifidobacterium adolescentis*, as one of the core gut symbionts, has garnered significant attention in recent years for its potential in alleviating FC. Preliminary studies suggest this bacterium can ferment dietary fiber to produce SCFAs such as acetate and butyrate. This lowers intestinal pH and stimulates the myenteric plexus, enhancing colonic motility and mucus secretion [26]. Consequently, transit time is reduced and stool consistency is softened. In 2023, Xi’an Jiaotong University’s double-blind randomized controlled trial involving 250 participants demonstrated that a composite probiotic containing *Bifidobacterium* significantly reduced Bristol stool scores in constipated patients, accompanied by decreased plasma 5-HT levels. This suggests it improves defecation difficulties via the microbiota-enteric nervous system-hormone axis [27]. Further monostrain research on *B. adolescentis* yielded additional progress: a 2025 report identified strain *B. adolescentis XA-1434* as carrying the arabinoxylan glucanase *GH8* gene, enabling efficient degradation of arabinoxylan into oligosaccharide by-products [28]. In constipated mouse models, this significantly enhanced small intestinal propulsion rate and 6-h fecal output in a dose-dependent manner [29]. This study first established the chained relationship between strain-specificity, carbon source utilization, and motility improvement, providing a basis for precise strain selection. Additionally, animal experiments demonstrated that *B. adolescentis* upregulates the c-Kit/SCF signaling pathway in the colon, restoring the Cajal interstitial cell network and ameliorating slow-transit constipation [30]. Concurrently, it alleviates low-grade inflammation by elevating IL-10 and reducing TNF-α, thereby indirectly decreasing visceral hypersensitivity [31]. In summary, *B. adolescentis* may alleviate functional constipation through a triple synergistic mechanism involving SCFA-neural excitation, mucus-barrier repair, and immune-anti-inflammatory pathways. Possessing strain-specific functional gene advantages, its efficacy and optimal dosage warrant further validation in high-quality randomized controlled trials [32]. Metagenomic analysis revealed that prolonged spaceflight elevated the abundance of harmful bacteria such as *E. coli* and *K. oxytoca* while significantly reducing *B. adolescentis* and the butyrate-producing *Butyricimonas hominis*. This was accompanied by decreased glycoside hydrolases/transferases, abnormal activation of carbohydrate signaling pathways, and membrane transport pathways, collectively contributing to slow-transit constipation. This identified *B. adolescentis* as the key missing bacterium. Mechanistically, this study first elucidates how *B. adolescentis* alleviates post-long-haul constipation through three interacting pathways: (1) The arginine-urea cycle axis: colonization significantly upregulates colonic *Ugt1a1*, *Gpt*, and *Otc* expression in the colon (increased by 2.1, 1.8, and 2.3 fold, respectively; FDR < 0.01), accelerating ornithine-polyamine synthesis, restoring nitric oxide homeostasis, and promoting Lgr5^+^ intestinal stem cell proliferation, thereby accelerating mucosal renewal [33]. (2) Bile acid metabolism axis: The expressions of *Ces1d*, *Reg3g* and *Ace2* have significantly increased, reversing TUDCA/TDCA depletion in constipated mice. Enhanced TGR5-MLCK signaling strengthened ZO-1/Claudin-1 tight junctions, reducing intestinal permeability by 28% [34]. (3) Barrier-lubrication restoration: Cup-shaped cell count increased by 42%, and mucus layer thickness rose by 35%, reducing excessive water reabsorption. Concurrently, strain-produced acetate lowered intestinal pH by 0.4 units, inhibiting *E. coli* and *K. oxytoca* colonization to establish a beneficial microbial community displacement effect. These three synergistic pathways enhance intestinal motility and barrier integrity, providing a mechanistic rationale for *B. adolescentis* as a precision probiotic formulation [35].

The disorder of intestinal flora structure is the reason for constipation after long-distance voyages. Regulating the balance of intestinal flora and improving the intestinal environment are the keys to the treatment of intestinal diseases. Chen (2024) found that the intake of Animal *Bifidobacteria* can increase the defecation frequency of children and improve constipation symptoms [14]. Meanwhile, it was found that *Bifidobacterium longum* can improve constipation by increasing the abundance of lactic acid bacteria in the intestine [36]. Dong (2023) demonstrated that the abundance of *Clostridium* was decreased in patients with constipation, and dietary fiber derived from litchi pulp combined with phenolic complexes could increase the abundance of this bacterium [37]. Wang (2019) showed the effectiveness of *B. adolescentis* in the treatment of loperamide hydrochloride-induced constipation in mice [38]. It is consistent with our conclusion, but whether there is specificity of *B. adolescentis* in the treatment of constipation remains to be studied [39]. In this study, the constipation model was established by bacteria suspension after a long-distance voyage, which is more likely to simulate the intestinal model of the constipation population and to verify and study the mechanism of *B. adolescentis* at the intestinal flora level.

Existing research suggests that the efficacy of *B. adolescentis* in alleviating FC exhibits a “strain-dose-host” dependency, with its mechanisms not yet fully elucidated. Regarding positive evidence, Wang et al. demonstrated in a mouse model that both *CCFM 669* and *CCFM 667* strains can perform the following: rapidly proliferate and adhere to the intestinal epithelium, increasing fecal propionic and butyric acid concentrations and enhancing small intestinal propulsion and shortening the time to first black stool passage, thereby significantly improving loperamide-induced constipation. This was accompanied by increased *Lactobacillus* and reduced *Clostridium*, suggesting action via the “acidification of the microenvironment-modulation of the microbiota-stimulation of motility” axis [40]. However, the same study also observed that high doses of *CCFM 626* were ineffective, with only low doses exhibiting a mild laxative effect. This indicates that “greater bacterial load ≠ better efficacy,” suggesting the existence of a dose threshold and strain specificity [41]. Notably, some human trials yielded contrary conclusions: Favretto et al. reported that *Bifidobacterium* supplementation prolonged total intestinal transit time, suggesting that *Bifidobacterium adolescentis* may produce conflicting physiological effects under different host backgrounds or substrate conditions. In summary, *Bifidobacterium adolescentis* may alleviate constipation through pathways such as SCFA production, enhanced peristalsis, and suppression of potential pathogens [42]. However, its efficacy is multifactorially regulated by strain genetics, adhesion capacity, fermentation substrates, and baseline host microbiota status. Future studies requiring multi-omics and longitudinal clinical designs are needed to clarify under which conditions its mechanisms predominate or whether reverse inhibition of peristalsis may occur. This study not only verified that *B. adolescentis* can treat constipation by increasing 5-HT4R and downregulating membrane protein signaling. In the study of Wu (2019), hesperidin can relieve constipation symptoms by affecting the 5-HT4R/c-AMP signaling pathway [43]. c-AMP level and calcium ion concentration can affect each other. At the same time, calcium ion concentration can affect the movement of intestinal smooth muscle, increase fecal water content, and regulate neurotransmitters to affect constipation symptoms [44]. Wen (2024) showed that total flavonoids of *Fructus aurantii Immaturus* alleviated constipation by alleviating the calcium ion concentration balance in interstitial cells of Cajal through frizzled-2 [45]. *Slc8a1* is a transmembrane transporter that can transport neurotransmitters in the gut to synaptic vesicles, promote the transport of 5-HT4R between cells, realize the binding with G protein-coupled receptors, regulate intracellular and extracellular Ca^2+^ exchange, and promote intestinal motility to treat constipation [46]. This study also discovers the appearance of constipation affects the signaling pathways related to membrane transport and signaling, which is consistent with the results of previous studies. Therefore, this research states that the occurrence of constipation is highly related to carbohydrate metabolism, membrane transport, signaling, and so on. The mechanism of constipation can be studied and analyzed in the future.

Transcriptomic analysis indicates that *B. adolescentis* downregulates the membrane transport gene *Slc8a1*, thereby reducing extracellular calcium efflux. This enhances 5-HT4R membrane localization and elevates cAMP levels, leading to increased circular muscle contraction and a 32% reduction in the time to first black stool passage. Combined multi-omics analysis revealed that *B. adolescentis* could upregulate arginine metabolic pathways (*Ugt1a1*, *Gpt*, and *Otc*) and bile acid metabolic pathways (*Ces1d*, *Reg3g*, and *Ace2*) and increase the number of goblet cells to restore intestinal barrier function. Goblet cells are responsible for the production of mucus that protects the intestinal barrier and improves intestinal lubrication [47]. The decrease in the number of goblet cells leads to insufficient secretion of intestinal mucus, prolongs the residence time of feces in the intestine, and promotes excessive reabsorption of water, thus reducing the frequency of defecation and prolonging the time of the first case of melena [48]. In recent years, multicenter studies have successively demonstrated *Lactobacillus acidophilus ATCC314*, *B. adolescentis CCFM667*, and *L. plantarum agrC/A* deletion strain can reverse FXR suppression induced by a high-fat diet or voyage stress via the bile acid-FXR-FGF15/19 signaling pathway. This reduces hydrophobic secondary bile acids such as DCA/LCA, restores *CYP7A1* negative feedback, and consequently ameliorates hepatic steatosis and colonic motility [49,50,51]. Arginine is produced in the human liver and is involved in protein metabolism and the urea cycle. Xie (2021) found that the content of arginine in the serum of patients increased after treatment [52]. In recent years, studies have found that arginine plays an important role in the proliferation and development of intestinal stem cells [53]. Wu (2020) found that intake of 0.4% arginine could promote the production of intestinal endocrine immunoglobulin in mice [54]. Studies have demonstrated that compared with healthy people, the content of fecal bile acids and primary bile acids in patients with constipation is significantly reduced [55]. According to the existing studies, it can be proved that the content of bile acids is correlated with the occurrence of constipation symptoms. As a gene of the carboxylesterase family, *Ces1d* has the function of protecting the liver and can catalyze the lipid hydrolysis reaction related to bile acid metabolism. The reduction of its abundance causes liver damage and then affects bile acid metabolism. *Ace2* and *Reg3g* have the protective function of intestinal barrier, preventing harmful bacteria from entering the intestinal blood and preventing the occurrence of inflammation [56,57]. This study confirms the gut microbiota biomarkers of long-distance travelers. Transcriptomic analysis demonstrates that *B. adolescentis* can alleviate constipation by upregulating the arginine metabolic pathway and bile acid metabolic pathway, thereby effectively increasing intestinal barrier proteins and enhancing intestinal barrier function.

Meanwhile, studies have shown that *Ace2* can regulate the composition of intestinal flora and affect the 7 α-dehydroxylation process of bile acids. Some bacteria (such as *Lactobacillus* and *Clostridium*) are responsible for converting primary bile acids into secondary bile acids [58,59]. It has been shown that *Ace2* knockout mice exhibit increased bile acid content and liver injury [60]. At the same time, the deletion or inhibition of *Reg3g* will lead to the imbalance of the intestinal microbial community and the impairment of intestinal barrier function [57]. After targeted bile acid metabolomic analysis, it was found that the contents of tauroursodeoxycholic acid (TUDCA) and taurodeoxycholic acid (TDCA) were significantly decreased in constipated mice. In Lien et al., it was found that the intestinal validation response was reduced after tduca supplementation [61]. Similarly, TUDCA can improve the intestinal barrier function associated with the TGR5-MLCK pathway to restore intestinal health [62]. TDCA has proved to have anti-inflammatory and protective effects on the intestine, as well as regulating the Nrf2-mediated signaling pathway to alleviate intestinal oxidative stress response to improve intestinal barrier health [63]. This study, combined with previous studies, showed that the occurrence of constipation cannot be separated from the disturbance of bile acid and arginine metabolism, resulting in the impairment of the intestinal barrier. ZO-1 and Claudin-1 jointly maintain the tight junction of intestinal epithelial cells and maintain the intestinal barrier function. It is verified that *B. adolescentis* can effectively increase intestinal barrier protein and intestinal barrier function by improving arginine and bile acid metabolism. Bile acid metabolism module: *Ces1d*, *Reg3g*, and *Ace2*.

In this study, metagenomic analysis was carried out on the feces of long-distance voyagers before and after the voyage to explore the causes of constipation symptoms after the voyage and to find the bacteria for its treatment, and the bacteria were verified by animal experiments, and the mechanism of *B. adolescentis* in the treatment of constipation after the voyage was found through a variety of omics joint analyses. The results showed that after the long voyage, the abundance of harmful bacteria such as *Escherichia coli* and *Klebsiella oxytoca* increased, while the abundance of beneficial bacteria such as *B. adolescentis* and *butyricimonas hominis* decreased, which led to the decrease in the abundance of glycoside hydrolases and glycosyltransferases, affected the carbohydrate-related signaling pathways, caused the increase in the abundance of signaling pathways related to membrane transport and signaling, and finally led to the occurrence of constipation disease after the long voyage. *B. adolescentis* can upregulate arginine metabolic pathways (*Ugt1a1*, *Gpt*, and *Otc*) and bile acid metabolic pathways (*Ces1d*, *Reg3g*, *Ace2*) and increase the number of goblet cells to restore intestinal barrier function. It can also downregulate membrane protein signaling, involving *Slc8a1* and other genes, to increase the expression of intestinal 5-HT4R protein and improve intestinal motility to treat constipation symptoms after long-distance voyages. In conclusion, this study established standardized techniques for fecal microbiota transplantation and conducted functional research on biomark bacteria for constipation following long-distance voyages. This work provides a scientific basis for exploring microbial functions and functional health, and will further deepen our understanding of the impact of the gut microbiota on human health.

This study demonstrates through multi-omics research that *Bifidobacterium adolescentis* can regulate bile acid metabolism and improve colonic mucosal integrity in constipation models via a calcium ion-mediated barrier repair mechanism.

This study verifies that *B. adolescentis* can effectively relieve constipation symptoms after long-distance voyages, provide help for solving constipation symptoms after long-distance voyages, and provide research support for the research and development of probiotics. While this study demonstrates that *B. adolescentis* alleviates constipation through modulation of bile acid and arginine metabolism pathways, our analysis was limited to bile acid quantification. To fully elucidate the mechanistic basis, future studies should incorporate the following: (1) comprehensive arginine metabolic profiling and (2) validation of bile acid-arginine crosstalk in animal models, thereby strengthening the evidence for probiotic efficacy in constipation treatment. The sample size of this study is limited, and there is a lack of clinical cohort verification. In the future, it is necessary to expand the number of participants and conduct randomized controlled trials to further confirm the efficacy and safety of *B. adolescentis* in the treatment of post-sea travel constipation.

## 4. Materials and Methods

A total of 73 stool samples were collected from male long-distance voyagers after 6 months, of which 43 were from pre-voyage and 30 were collected after long-distance voyage. Donor number and selection criteria: A total of 73 fecal donors (all male) were included, with an age range of 22–35 years (average 27.4 ± 3.2 years) and a BMI of 19.5–24.2 kg/m^2^ (average 21.8 ± 1.6 kg/m^2^). All donors were long-term residents of the East China region (≥5 years), with no history of migration between the north and south. All donors had no history of gastrointestinal diseases, metabolic disorders, mental illnesses, or use of antibiotics/probiotics (at least 3 months ago), and their health was confirmed through questionnaire surveys. Fecal samples were immediately placed in an anaerobic transport bag after collection, transferred to the laboratory within 4 h, aliquoted, and frozen at −80 °C until DNA extraction. This information has been added to the “Fecal Sample Collection and Storage” section to ensure the reproducibility of the experiment and the reliability of the data. All donors agree to complete the Sample Collection Information Form and the Sampling Know-You Form.

### 4.1. Metagenomic Sequencing and Taxonomic Analysis

Total DNA was extracted from the collected stool samples for sequencing analysis, and DNA extraction was performed according to the kit instructions. Libraries were then constructed using the ruSeq Nano DNA LT Sample Preparation Kit (Illumina, San Diego, CA, USA). The sequencing and data analysis part of the database was completed by Shanghai Ouyi Biomedical Technology Co., Ltd. (Shanghai, China). The libraries were sequenced using the Illumina Novaseq 6000 sequencing platform at the Shanghai OE Biotech Co., Ltd (Shanghai, China) sequencing platform, followed by fastp (v 0.20.1) to delink the raw off-the-fly data (FastQ file) and filter out the low-quality bases to remove the N-bases (fuzzy bases) reads. It was aligned with the host genome, and the host sequence was removed. MMSeqs2 (v 13.45111) was used to construct the non-redundant gene set of the predicted genes in all samples, and the longest gene in each cluster set was selected as the representative sequence of the gene set, and the clean reads of each sample were compared with the non-redundant gene set (95% identity) by Salmon (v 1.8.0), and the abundance information of genes in the corresponding samples was counted. Species annotations were obtained from the taxonomic information database corresponding to the NR library, and then the abundance of that species was synthetically calculated using the gene abundance corresponding to the species. The abundance of species in each sample was counted at the taxonomic level of phylum, family, genus, and species, and the representative sequences of the gene set were compared and annotated with KEGG and CAZy data by DIAMOND (v 0.9.10.111) software, and the expected e-value of BLAST (v 2.14.0) alignment parameters was set to 1 × 10^−5^. PCoA analysis and mapping of species using the R package (v 4.1.2). Based on the R package, the difference analysis was performed using Wilcoxon test (https://search.r-project.org/CRAN/refmans/rstatix/html/wilcox_test.html (accessed on 7 December 2025)). LEfSe was used to analyze the differences in species abundance profiles or functional abundance profiles (LDA ≥ 3.5). Genomic DNA was extracted from samples using the MagPure Soil DNA LQ Kit from Magen Biotechnology Co., Ltd (Guangzhou, China). Library preparation was performed with the VAHTS Universal PLUS DNA Library Prep Kit by Vazyme Biotechnology Co., Ltd (Guangzhou, China), involving enzymatic fragmentation and end-repair of DNA fragments, followed by Illumina sequencing adapter ligation. The ligation products underwent magnetic bead purification and dual-wheel sorting to enrich target fragments. PCR amplification was performed using index-specific primers (i5/i7), with cycle numbers determined by the initial DNA quantity (5–100 ng). The final amplified library underwent magnetic bead purification, followed by quality control for concentration and fragment distribution using the Qubit dsDNA HS Assay by Thermo Fisher Scientific, Inc. (Waltham, MA, USA) and Agilent 2100 Bioanalyzer by Agilent Technologies, Inc. (Santa Clara, CA, USA), respectively. Libraries passing quality control undergo high-throughput sequencing on the Illumina platform.

### 4.2. B. adolescentis Preparation

*Bifidobacterium* were isolated and cultured from stool samples prior to long-term voyages [64]. The stool sample was diluted with sterile normal saline at a ratio of 1:10, mixed well, and the supernatant was collected after centrifugation at 600 rpm. The supernatant was diluted to 10^−4^. We gather 0.1 mL of bacterial solution was incubated in MRS solid medium containing 1% L-cysteine and mupirocin salt at 37 °C for 24 h. Many suspected colonies with smooth, watery edges, white, or milky white were selected for microscopic examination, and the clearly bifurcated colonies were streaked and cultured in MRS solid medium containing 0.1% L-cysteine at 37 °C for 48–72 h. Single colonies were selected for Gram staining observation, and colonies with positive Gram staining were selected for further isolation.

DNA was extracted using the Ezup Column-based Bacterial Genomic DNA Extraction Kit, followed by PCR amplification of the raised DNA using the following primers: F:5′-GGTGTGAAAGTCCATCGCT-3′, R:5′-GTCTGCCAAGGCATCCACCA-3′ [65]. The amplified products were subjected to 1.5% agarose gel, 1× TAE electrophoresis, 150 V, and 100 mA for 20 min, and the electrophoresis results were observed. At the same time, the amplification products were sequenced and analyzed [66].

The single colonies that had been cultured for 48 h were inoculated in liquid MRS medium for 20 h at 37 °C, passaged twice at 3% inoculum amount, then anaerobically incubated at 37 °C for 24 h, centrifuged at 4 °C at 6000 r/min for 15 min, and resuspended in 100 g/L sterile skim milk until the cell density was 6 × 10^9^ CFU/mL. Aliquot according to the amount needed each time and store at −80 °C. It is used in a 37 °C water bath for 15 min to gavage [67].

### 4.3. Fecal Microbiota Transplantation (FMT) Preparation

The feces were added to sterile normal saline at a ratio of 1:10, mixed well, and centrifuged at 4 °C at 6000 r/min for 15 min to collect the bacterial sludge, and the bacterial sludge was remixed with sterile normal saline. The bacterial *solution* was aliquoted in a 1:1 ratio into sterile skim milk, stored at −80 °C, and used in a 37 °C water bath for 15 min prior to gavage [68].

### 4.4. Animal Experiments

**Figure 8:**
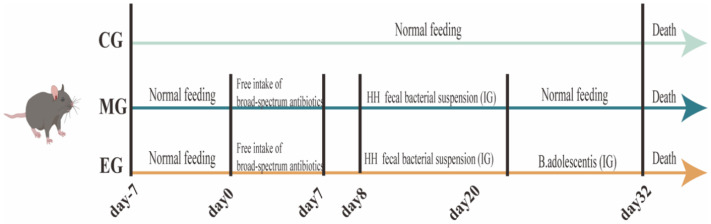
Flow chart of animal model establishment. CG, MG, and EG were the normal control group, model group, and experimental group, respectively. IG indicates oral gavage, and HH indicates the use of long-term post-flight stool for experiments.

The animal experiment was approved by the Animal Ethics Committee of the Naval Medical University. Approval number: CHEO2024-122. Purchased 6-week-old male featureless pathogen (SPF) C57BL/6J mice from Shanghai Just-Wise Laboratory Animal Co. Ltd. (Shanghai, China). After one week of acclimatization, 24 mice were grouped into the normal control group (CG), the post-navigation fecal suspension group (MG), and the *B. adolescentis* treatment group (EG), with 8 mice in each group. The experimental design is shown in Figure 8. Except for CG, the other groups were given 7 days of 1 g/L of metronidazole, neomycin sulfate, ampicillin sodium, and 0.5 g/L of vancomycin hydrochloride [69] for free drinking water. Starting from the 8th day, mice in groups MG and EG were given 0.2 mL of post-flight fecal bacteria suspension by oral gavage for 12 consecutive days. Subsequently, mice in EG were given an oral gavage of 0.2 mL of *B. adolescentis* [70]. After 12 days of modeling, colon tissue from each group was collected for follow-up experiments.

### 4.5. Animal Experimental

Record of mouse defecation quality, water content, and shape. During the experiment, the parameters of mouse defecation within 2 h after successful modeling were recorded, including fecal frequency, fecal weight, fecal dry weight, and fecal water content. Mice were housed individually in cages, reared with sufficient food and water, and fecal weights were measured within 2 h, and then fecal dry weights were determined after feces were dried at 75 °C for 24 h. The fecal moisture content was calculated. Fecal wet weight Fecal dry weight:

$$\mathrm{Fecal}\;\mathrm{moisture}\;\mathrm{content}\;=\;\frac{\mathrm{Fecal wet weight}-\mathrm{Fecal dry weight}}{\mathrm{Fecal dry weight}}\times 100\%$$

Time and fecal frequency of the first appearance of melena in mice: Before the experiment, the mice were fasted for 12 h, and each mouse was given 0.2 mL of Indian ink gavage, and the time of the first melena and the number of bowel movements within 4 h were recorded.

### 4.6. Histopathological Staining Analysis

The collected mouse colon tissue was fixed in 4% paraformaldehyde for 24–48 h, followed by routine ethanol gradient dehydration, xylene clearing, and paraffin embedding. Paraffin sections 4–5 μm thick were prepared using a paraffin microtome, followed by dewaxing and rehydration prior to hematoxylin and eosin (H&E) staining. Following ethanol gradient dehydration and xylene clearing, stained sections were mounted with neutral resin. All sections were examined under light microscopy by two researchers unaware of group assignments. Images were randomly captured using a digital imaging system for semi-quantitative assessment of specific pathological alterations. This experiment was conducted by Sevier Corporation.

### 4.7. Immunohistochemistry

For immunohistochemical analysis, sections were subjected to antigen retrieval in citrate buffer (pH 6.0) following dewaxing and rehydration. Endogenous peroxidase activity was subsequently blocked with 3% hydrogen peroxide solution, followed by blocking at room temperature in 5% bovine serum albumin. Primary antibody incubation was performed overnight (4 °C) using a 1:200 dilution of anti-5-HTR4 antibody, followed by a 50 min incubation at room temperature with HRP-conjugated goat anti-rabbit IgG. Color development was performed using a DAB color development kit by Beyotime Biotech Inc. (Shanghai, China), followed by hematoxylin counterstaining of nuclei and mounting. All sections were examined by two researchers unaware of the group assignments under an optical microscope. Images were randomly captured using a digital imaging system and analyzed with image processing software (ImageJ 1.52).

### 4.8. Transcriptomic Analysis

The total RNA of colon tissues was randomly collected from the CG, MG, and EG (3 animals in each group), and then the libraries were constructed, and the libraries were sequenced using the Illumina Novaseq 6000 sequencing platform, and 150 bp paired-end reads were generated. HISAT2 (v 2.2.1) software performed reference genome alignment, gene expression (FPKM) calculations, and reads counts for each gene were obtained by HTSeq-count. PCA analysis of genes (counts) using R (v 3.2.0) and plotting to assess sample biological replicates. DESeq2 (v 3.22) software was used to analyze differentially expressed genes, and the differential genes were screened with q value of <0.05 and foldchange > 2 or foldchange < 0.5, and the enrichment analysis of differentially expressed genes was carried out based on the hypergeometric distribution algorithm to screen the significant enrichment functional items. R (v 3.2.0) was used to plot the significant enrichment function items.

RT-qPCR was used to determine the expression levels of related genes in mouse colon tissues. RNA was extracted and purified from the colon tissues of each group of mice using RNA extraction kits, and reverse transcribed into cDNA according to the reverse transcription instructions. Primers in the table were designed on NCBI and prepared by Sangon Biotech. The relative expression levels of *Ces1d*, *Otc*, *Ugt1a1* and *Reg3g* mRNA in mouse colon tissues were determined by real-time quantitative gene amplification. *GAPDH* was used as the reference gene. Details of each primer are shown in Table 4. Perform the experiment according to the chamQ Universal SYBR Master Mix manual.

### 4.9. Western Blot Analysis

Western Blot analysis was performed as follows: Tissue or cell samples were collected and total proteins extracted using RIPA lysis buffer (containing protease inhibitors). Following quantification by the Boiling Carboxymethylated Cellulose (BC) method, equal amounts of protein (20–40 μg) were subjected to SDS-PAGE electrophoresis and wet-transferred onto PVDF membranes. Following transfer, membranes were blocked with 5% skimmed milk and incubated overnight at 4 °C with primary antibodies (actin/Z-1/clathrin-1). Secondary antibodies conjugated to horseradish peroxidase (HRP) were then applied (2 h, room temperature). After final washing with TBST, signals were detected and visualized using ECL chemiluminescent reagents. Target band gray values were analyzed using ImageJ software, with relative expression levels expressed as the gray value ratio of the target protein to the internal control GAPDH.

### 4.10. Targeted Metabolomics Analysis

The sample was ground with methanol and centrifuged to collect the supernatant. Chromatographic analysis was performed under the following conditions: injection volume, 5 μL; flow rate, 0.45 mL/min; mobile phase A, 0.1% formic acid in water; mobile phase B, a mixture of methanol, acetonitrile, and isopropanol (1:1:1, *v*/*v*/*v*) containing 0.1% formic acid. The gradient elution program was set as follows: 0–0.5 min, 80% A/20% B; 0.5–1.5 min, 80% A/20% B; 1.5–12 min, 62% A/38% B; 12–17.5 min, 50% A/50% B; 17.5–19 min, 5% A/95% B; 19–19.01 min, 80% A/20% B; and 19.01–20 min, 80% A/20% B. Subsequent mass spectrometric analysis was conducted with the following parameters: curtain gas, 35 psi; collision-activated dissociation (CAD) parameter, medium; ion spray voltage, −4500 V in negative ion mode and 5500 V in positive ion mode; ion source temperature, 450 °C; column temperature, 45 °C; nebulizer gas (Gas1), 55 psi; and auxiliary heating gas (Gas2), 55 psi.

## Figures and Tables

**Table 1:** Comparison of indices by group in diversity analysis.

Classify	Chao1	Goods Coverage	Shannon	Simpson	ACE
HQ	5579.69 ± 635.12	1.00 ± 0.00	3.99 ± 0.56	0.91 ± 0.06	5488.88 ± 622.68 ^a^
HH	5073.90 ± 724.64	1.00 ± 0.00	3.86 ± 0.65	0.90 ± 0.07	4980.38 ± 716.77

^a^: HQ A is significantly different from HH (*p* < 0.05).

**Table 2:** List of pathway-enriched genes.

Gene	GO Enrichment Analysis	KEGG Enrichment Analysis
*Arg2*, *Otc*, *Nags*	Urea cycle	Amino acid metabolism
*Ugt1a1*, *Ugt1a6a*, *Ugt1a7c*		Carbohydrate metabolism
		Metabolism of cofactors and vitamins
		Xenobiotics biodegradation and metabolism
*Ces1d*, *Ces1f*	Regulation of bile acid secretion	Xenobiotics biodegradation and metabolism
	Retinyl-palmitate esterase activity	
	regulation of bile acid biosynthetic process	
	Positive regulation of cholesterol metabolic process	
	Negative regulation of cholesterol storage	
	Carboxylic ester hydrolase activity	
*Reg3b*, *Reg3g*, *Retnlb*	extracellular space	
	Extracellular region	
*Slc22a4*, *Slc51a*, *Slc51b*	Plasma membrane	Digestive system
	Transmembrane transporter activity	
	transmembrane transport	

**Table 3:** Content of various bile acids.

Metabolites	CG	MG	EG
Chenodeoxycholic Acid (CDCA)	1036.22 ± 439.47	1526.98 ± 1356.83	1148.00 ± 852.88
Deoxycholic Acid (DCA)	333,286.50 ± 169,045.41	198,666.02 ± 187,157.48	214,611.41 ± 183,025.15
Deoxycholic Acid Glycine Conjugate (GDCA)	50.21 ± 59.72	60.17 ± 78.13	51.33 ± 64.76
Tauroursodeoxycholic Acid (TUDCA)	658.75 ± 449.61	240.99 ± 69.24 ^a^	1028.01 ± 2247.92
Taurodeoxycholic Acid (TDCA)	1268.50 ± 407.62	311.65 ± 213.09 ^a^	1683.89 ± 3066.63
Taurochenodesoxycholic Acid (TCDCA)	465.21 ± 353.45	334.17 ± 345.71	3977.52 ± 9881.12
Taurocholic Acid (TCA)	8580.05 ± 7647.70	6216.21 ± 9382.47	71,555.80 ± 178,947.53
Hyodeoxycholic Acid (HDCA)	7102.37 ± 2967.93	14,535.80 ± 7268.79 ^a^	13,099.94 ± 8326.73
12-Ketolithocholic Acid (12-KLCA)	37,967.41 ± 20,635.87	52,149.39 ± 36,946.11	40,606.88 ± 23,839.74
Ursocholic Acid (UCA)	487.52 ± 617.71	1612.95 ± 2230.64	1443.59 ± 1062.88 ^a^
Allocholic Acid (ALCA)	2367.74 ± 2417.41	4339.47 ± 5141.82	3078.98 ± 2444.52
Dehydrolithocholic acid	810.25 ± 698.29	2830.01 ± 1983.22 ^a^	1787.67 ± 711.81 ^a^
3Beta-deoxycholic acid	17,075.30 ± 7851.22	13,295.99 ± 13,160.59	12,774.56 ± 10,877.11
3-Oxocholic acid	3145.81 ± 2496.71	9987.35 ± 12,642.66	19,945.37 ± 25,903.83
Cholic acid 7 sulfates	209,175.45 ± 219,268.70	331,803.29 ± 198,851.27	306,305.34 ± 355,695.34

^a^: Significant difference between the CG group and the MG group. (*p* < 0.05).

**Table 4:** Primer sequences of genes.

NCBI Number	Gene	Sequence (5′-3′)	Tm (°C)	Size (bp)
NM.053200.2	*Ces1d*	F:5′-GCCAACTTTGCTCGGAATGG-3′	60	104
		R:5′-GCCTGAGTTGAGGCACCAAT-3′		
NM.001411675.1	*Otc*	F:5′-AGATCTCACCATGCCCCTTG-3′	60	82
		R:5′-GCATAGCCCTCCCTTTGGAA-3′		
NM.201645.2	*Ugt1a1*	F:5′-TGGGAGGCTGTTAGTGTTCC-3′	60	102
		R:5′-GCTATGACCACAACTTCGTGC-3′		
NM_001411840.1	*GAPDH*	F:5′-AGGTCGGTGTGAACGGATTTG-3′	60	104
		R:5′-TGTAGACCATGTAGTTGAGGTCA-3′		
NM.011260.2	*Reg3g*	F:5′- CAGACAAGATGCTTCCCCGT-3′	60	94
		R:5′- GCAACTTCACCTTGCACCTG-3′		

## Data Availability

The original contributions presented in this study are included in the article/Supplementary Material. Further inquiries can be directed to the corresponding authors.

## Supplementary Materials

The following supporting information can be downloaded at: https://www.mdpi.com/article/10.3390/ijms262412142/s1.
