# Supplementary material for: Bifidobacterium adolescentis Strengthens Gut Barrier in Post-Voyage Functional Constipation
Source: Int J Mol Sci. 2025 Dec 17;26(24):12142. doi: 10.3390/ijms262412142 (PMC12734236; doi:10.3390/ijms262412142)
Supplement: Supplementary file 1 [file ijms-26-12142-s001.zip › ijms-3918004-supplementary.pdf]

B. adolescentis treats function constipation through Ca^{2+} mediated intestinal barrier repair

Attachment file S1

Analysis of functional differences before and after long-distance voyage

KEGG analysis of fecal bacteria before and after the voyage (S1A/B) showed that the main enriched functions of genes were related to metabolic pathway and environmental information processing pathway. Metabolic pathways mainly include global and overview maps, carbohydrate metabolism, amino acid metabolism, metabolism of cofactors and vitamins, and glycan biosynthesis and metabolism. Environmental information processing pathways are mainly membrane transport and signal transduction. In the tertiary pathways, the abundance of glycolysis / gluconeogenesis (ko00010), microbial metabolism in different environments (ko01120), citrate cycle (TCA cycle) (ko00020), calcium signaling pathway (ko04020), ABC transporters (ko02010) ABC transport, AMPK signaling pathway (ko04152) AMPK signaling pathway and other pathways increased after the voyage compared with that before the voyage. Compared with before the voyage, the abundance of pathways such as N-glycan biosynthesis (ko00510), various types of N-glycan biosynthesis (ko00513), and other glycan degradation (ko00511) decreased after the voyage. To sum up, after the long voyage, the abundance of pathways related to sugars decreased, and the abundance of pathways involved in membrane transport and signaling increased. It can be roughly inferred that the symptoms of constipation after long voyage are mainly related to the pathways of carbohydrate degradation, membrane transport and signaling.

According to Wilcoxon analysis, it can be found that the generation of long-term post flight constipation is mainly related to the pathways of carbohydrate degradation, membrane transport and signaling. The cazy function of faecal bacteria before and after the voyage was analyzed. It is mainly found that glycoside hydrolases, glycosyl transferases and carbohydrate esterases are related. Wilcoxon analysis showed that the abundance of GH1 (β-glucosidase), GH23 (chitosanase /chitinase), GT9 (cellulose synthase), AA1 (auxiliary activity family), GH37 (α-n-acetylgalactosaminidase), GH24 (N-acetyl-β-d-glucosaminidase), GH95 (α-fucosidase), gh29 (α-glucosidase), CE1 (carbonatase/acetylxylanase), GH28 (pectin lyase), GH20 (β-n-acetylglucosaminidase), GH92 (α-mannosidase the abundance of enzymes such as GT4 (cellobiose synthase) decreased significantly(S2 A/B/C). At the same time, the correlation analysis between the species at the genus level and cazy revealed that GH, GT and CE were all related to the decline in the abundance of *Bacteroides*, *phocaeicola*, *segatella* and *Prevotella* and the increase in the abundance of *Klebsiella*, *Escherichia* and *Citrobacter* after the flight. (S2 D)

It shows that the increase in the abundance of *Klebsiella*, *Escherichia* and *Citrobacter* and the decline in the abundance of *Ateroides*, *Phocaeicola*, *Segatella* and *Prevotella* caused by long-term voyage have led to the decline in the abundance of glycoside hydrolases, glycosyl transferases and carbohydrate esterases, affecting carbohydrate metabolism.

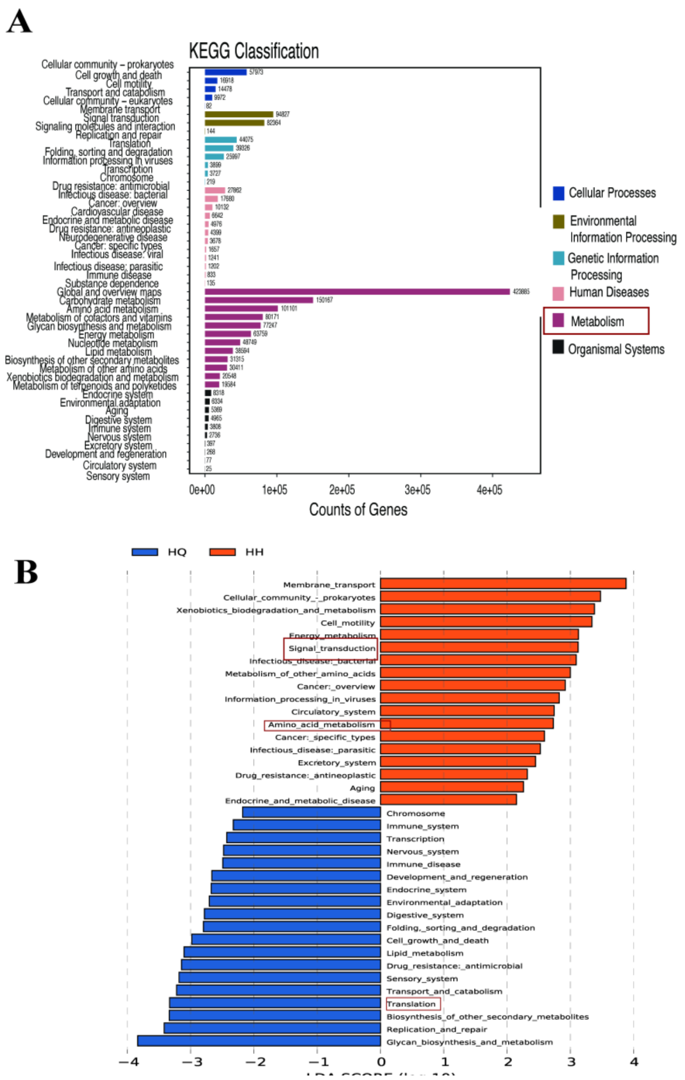

Figure S1. KEGG function annotation data analysis diagram. A shows the statistical chart of the number of annotated genes in KEGG database; B is the heat map of the difference between the two groups at KEGG level3 level.

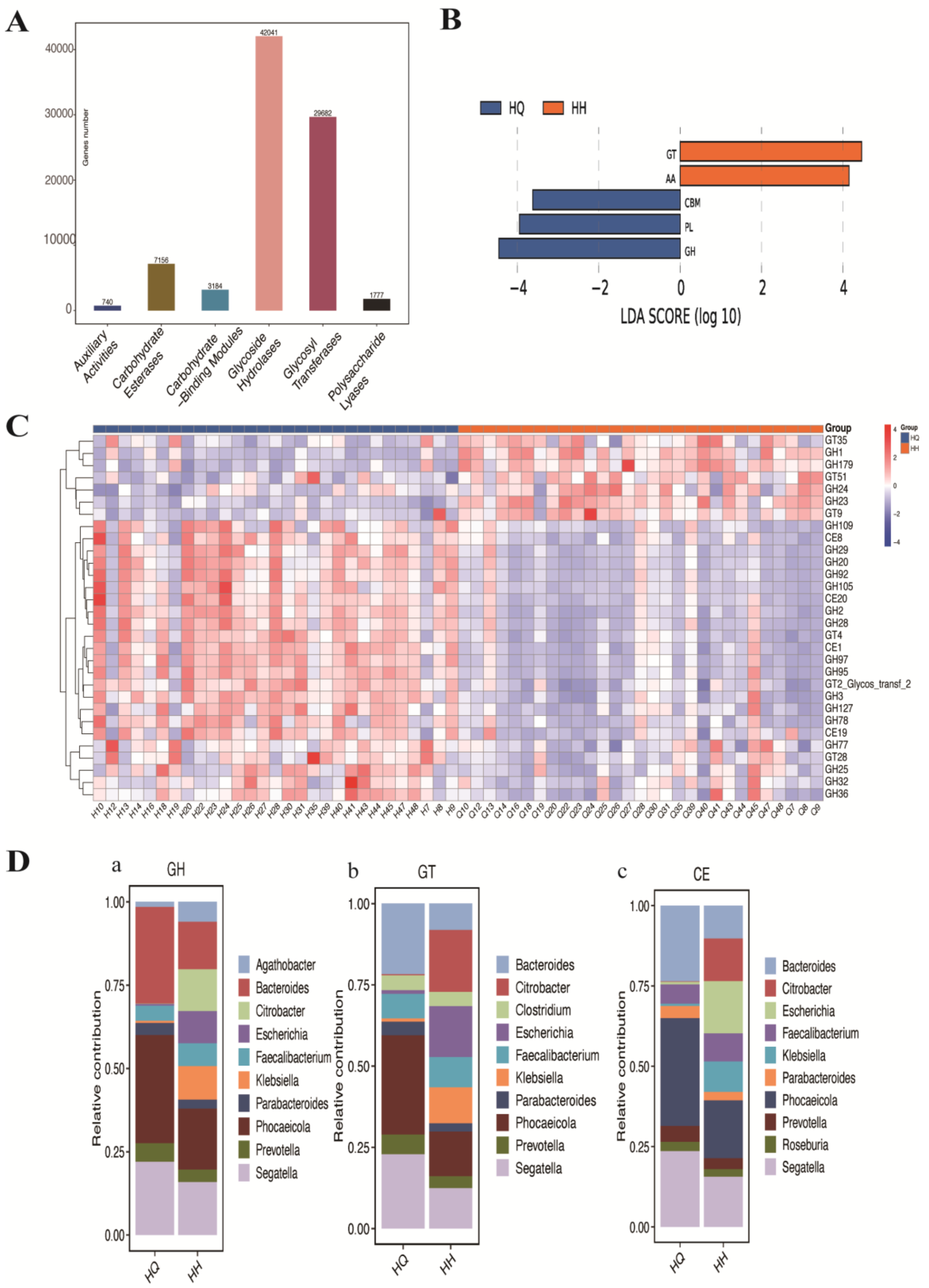

Figure S2. CAZy function annotation data analysis diagram. A shows the number of annotated genes in cazy database; B is the Heatmap diagram of differential function; C shows the lefse analysis atlas; D is the functional contribution analysis diagram between different enzymes and strains.
